# Editorial: Psychology, Technological Innovation, and Entrepreneurship

**DOI:** 10.3389/fpsyg.2019.02232

**Published:** 2019-10-10

**Authors:** Jesús de la Fuente, Douglas F. Kauffman, Unai Díaz-Orueta

**Affiliations:** ^1^Educational & Psychology School, University of Navarra, Pamplona, Spain; ^2^University of Almería, Almería, Spain; ^3^Medical University of the Americas, Devens, MA, United States; ^4^Department of Psychology, Maynooth University, Maynooth, Ireland

**Keywords:** psychology, R&D value chain, technological innovation, entrepreneurship, transference

The aim of this *Research Topic* is to offer an integrated view of three areas for implementing Psychology as a science and as a profession, for the benefit of both the academic and professional sphere. An initial article offers a global analysis of the R&D&I value chain (de la Fuente et al.). Complementarily, several articles then provide examples of *research* on the characteristics of Innovation and Entrepreneurship, whether as a review (Sánchez-García et al.), an analysis of a personal factor that is predictive of this activity (Arco-Tirado et al.), the role of psychological characteristics (Hu et al.), and even a tool for assessing this construct (Cuesta et al.).

Other articles document evidence of *technological development*. In primary education, evidence is presented about an app for learning mathematics (Mera et al.) and about a technological tool for assessing reading competence (Navarro et al.). In secondary education, we find evidence of online prevention of cyberbullying in adolescence (Garaigordobil and Martínez-Valderrey), as well as the characteristics and structure of an online tool for preventing alcohol intake in adolescence (de la Fuente et al.). In university education, we witness the effects of using technological tools during learning (Sáez-Manzanares et al.) and the use of another technological tool for assessing stress in university students (de la Fuente et al.). Finally, pure research has been applied to the field of intracranial stimulation for musical perception (Sánchez-Kuhn et al.).

In conclusion, other studies show examples of *transfer activities*, the central vision of Leadership and Entrepreneurship (Palazzeschi et al.), the effects of training for this activity in the workplace (Ho et al.) and a market study (Wan et al.).

## Author Contributions

JF had the idea and coordinated the Research Topic. DK and UD-O carried out support tasks for the coordination of the Research Topic and Edition of Articles.

### Conflict of Interest

The authors declare that the research was conducted in the absence of any commercial or financial relationships that could be construed as a potential conflict of interest.

